# Quantitative Proteomics and Phosphoproteomics Reveal TNF-α-Mediated Protein Functions in Hepatocytes

**DOI:** 10.3390/molecules26185472

**Published:** 2021-09-08

**Authors:** Rodrigo Mohallem, Uma K. Aryal

**Affiliations:** 1 Department of Comparative Pathobiology, Purdue University, West Lafayette, IN 47907, USA; ferreir@purdue.edu; 2 Purdue Proteomics Facility, Bindley Bioscience Center, Purdue University, West Lafayette, IN 47907, USA

**Keywords:** proteomics, insulin resistance, diabetes, hepatocytes, cell cycle

## Abstract

Increased secretion of proinflammatory cytokines, such as tumor necrosis factor-alpha (TNFα), is often associated with adipose tissue dysregulation, which often accompanies obesity. High levels of TNFα have been linked to the development of insulin resistance in several tissues and organs, including skeletal muscle and the liver. In this study, we examined the complex regulatory roles of TNFα in murine hepatocytes utilizing a combination of global proteomic and phosphoproteomic analyses. Our results show that TNFα promotes extensive changes not only of protein levels, but also the dynamics of their downstream phosphorylation signaling. We provide evidence that TNFα induces DNA replication and promotes G1/S transition through activation of the MAPK pathway. Our data also highlight several other novel proteins, many of which are regulated by phosphorylation and play a role in the progression and development of insulin resistance in hepatocytes.

## 1. Introduction

Tumor necrosis factor-alpha (TNFα) is primarily secreted by a hyperplastic and hypertrophic adipose tissue, typically associated with obesity [1]. The overexpression of TNFα also disrupts insulin signaling by decreasing insulin receptor (IR) autophosphorylation, dampening the signaling cascade, and culminating in decreased glucose uptake [2]. Although the molecular mechanisms underlying the regulatory effects of TNFα in adipocytes and adipose tissue during obesity are well-studied, there is little information about the molecular consequences of hepatocytes in response to TNFα exposure, despite its upregulation in obesity [3].

The direct stimulation of glucose uptake and metabolism is mediated by insulin. Insulin is the single hormone responsible for the regulation of blood glucose concentration, acting through activation of the canonical glycolytic pathway in target cells [4]. Insulin resistance occurs when normal circulatory insulin concentration become insufficient for promoting glucose uptake and subsequent intracellular glucose homeostasis [5]. The insulin resistance phenotype is primarily observed in skeletal muscle, liver, and in adipose tissue. Hepatic insulin resistance is, however, particularly concerning, as it leads to hyperglycemia [6].

Hepatocytes have been shown to be particularly sensitive to Free Fatty Acids (FFAs). Elevated circulatory levels of FFAs have been demonstrated to induce a dysregulation of the canonical insulin signaling pathway, diminishing the phosphorylation of insulin receptor substrates 1 and 2 (IRS1 and 2) [7]. Elevated levels of FFA in rat liver tissue have been shown to promote the activation of the IKK/IκB/NF-κB pathway in hepatocytes. The downstream activation of NF-κB induces the transcription of proinflammatory cytokines, including TNFα and interleukine-6 (IL-6) [8].

Characterizing the downstream effectors of TNFα signaling in hepatocytes and elucidating subsequent biological implications is fundamental for a systemic understanding of obesity-associated pathologies including the development of T2DM. In this study, we used global proteomics and phosphoproteomics approaches to identify key proteins that are significantly regulated by, or in response to, TNFα exposure. Our data reveal that TNFα inhibits fatty acid and lipid metabolism, likely contributing to the accumulation of FFAs, which further contribute to insulin resistance. Moreover, we show that cell cycle proteins are significantly regulated and activated by TNFα treatment. Our data suggest that TNFα acts as a stimulator for entry in G1/S of the cell cycle. These results introduce a novel perspective on the functional roles of TNFα on hepatocytes, and can serve as a future reference for the development of therapeutic targets aiming to alleviate hepatic insulin resistance.

## 2. Results

### 2.1. Time-Resolved Quantitative Proteomic Analysis

To date, the molecular implications of TNFα-mediated inflammation in hepatocytes remain largely unexplored. Previous reports have, however, suggested that TNFα induces a sharp decrease in glucose uptake within as little as 1 h of treatment. Indeed, we observed a significant decrease in glucose uptake in AML12 murine hepatocytes after 2 and 8 h of 10 ng/uL TNFα treatment, with a recovery of glucose uptake at 24 h of single TNFα treatment compared with 0 h of treatment (Figure S1A).

**Figure 1:**
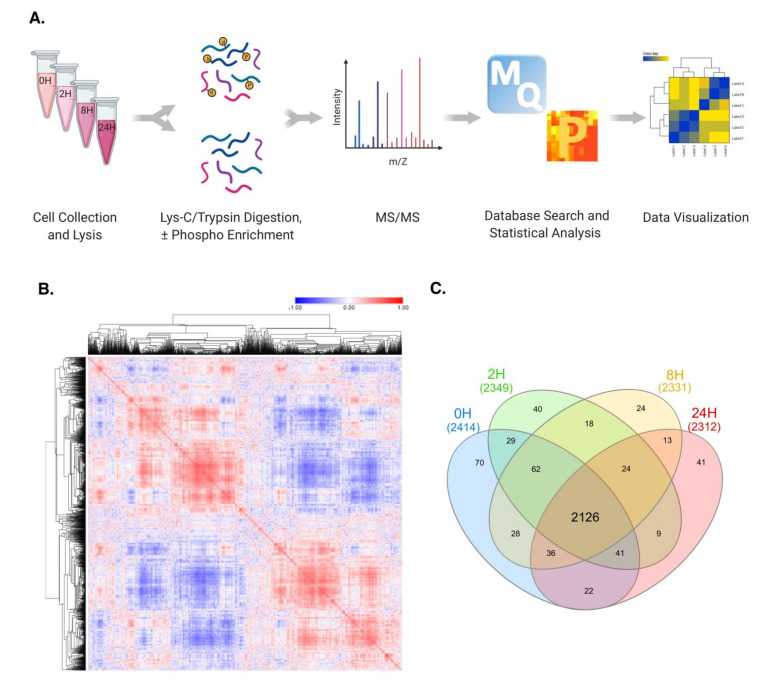
Global proteomic analysis of TNFα-treated murine hepatocytes. (**A**) Schematic representation of the experimental workflow. Cells were collected at 0H, 2H, 8H, and 24H, proteins were extracted and subsequently digested with Lys-C/Trypsin. A fraction of the total peptides was utilized for global proteomics, and the remainder was used for phosphoenrichment. Raw LC–MS/MS data were searched for with the MaxQuant software, and analyses were performed in the Perseus platform. (**B**) Pearson’s correlation profile of all quantified proteins. Red hue indicates higher correlation scores, while blue hue corresponds to lower correlation values. (**C**) Venn diagram representation of all quantified proteins identified in each time point. A total of 2126 proteins were quantified in all four time points, indicating a highly consistent data acquisition and sample reproducibility.

To explore the progressive effects of TNFα in the proteome of hepatocytes, AML12 murine hepatocytes were treated with 10 ng/uL TNFα and collected at 2 h (2H), 8 h (8H), and 24 h (24H) after initial treatment (0H). AML12 cells were subsequently homogenized and total protein was prepared as previously described [9]. A total 1 μg of peptides was used for global proteomic analysis, and the remainder was enriched for phosphopeptides with Polymer-based Metal-ion Affinity Capture (PolyMAC) spin tips (Tymora Analytical) prior to LC–MS/MS analysis. Raw LC–MS/MS data were searched for in the MaxQuant platform against the Uniprot *Mus musculus* database. Statistical analysis was then performed in the Perseus software [10,11] (Figure 1A).

In our global analysis, we identified a total of 3553 proteins, assigned from 33,704 peptides. From those, 2609 were considered quantified (LFQ > 0 in at least two biological replicates of the same time point). Quantified proteins only were used for the subsequent analyses presented herein, and represent the most comprehensive dataset to date for characterizing protein regulations in AML12 in response to TNFα (Tables S1 and S5).

### 2.2. TNFα Treatment Induces an Extensive Modulation of the Proteome Profile of Hepatocytes

To visualize our global data, while obtaining a large-scale perspective of the regulatory patterns of the proteins quantified, a correlation map of our global dataset was generated. Hierarchical clustering based on “one minus Pearson correlation” metric was applied, in which clear clusters were observed. The distinct areas of high and low correlation values indicate a noteworthy variance of LFQ values relative to the identified proteins (Figure 1B).

Despite the protein-dependent variations in LFQ values observed, 2126 of the 2609 proteins identified were quantified in all four time-points studied, indicating highly consistent data acquisition across all samples analyzed (Figure 1C). This consistency is further evidenced by the uniformity of LFQ value distribution evidenced by Violin plot analysis. The shape of each plot reveals a normal-like distribution of the data, again confirming the reliability of data acquisition (Figure S1B).

To explore the time-resolved molecular and biological ramifications of TNFα treatment, we focused on the statistically significant proteins curated from our global dataset.

**Figure 2:**
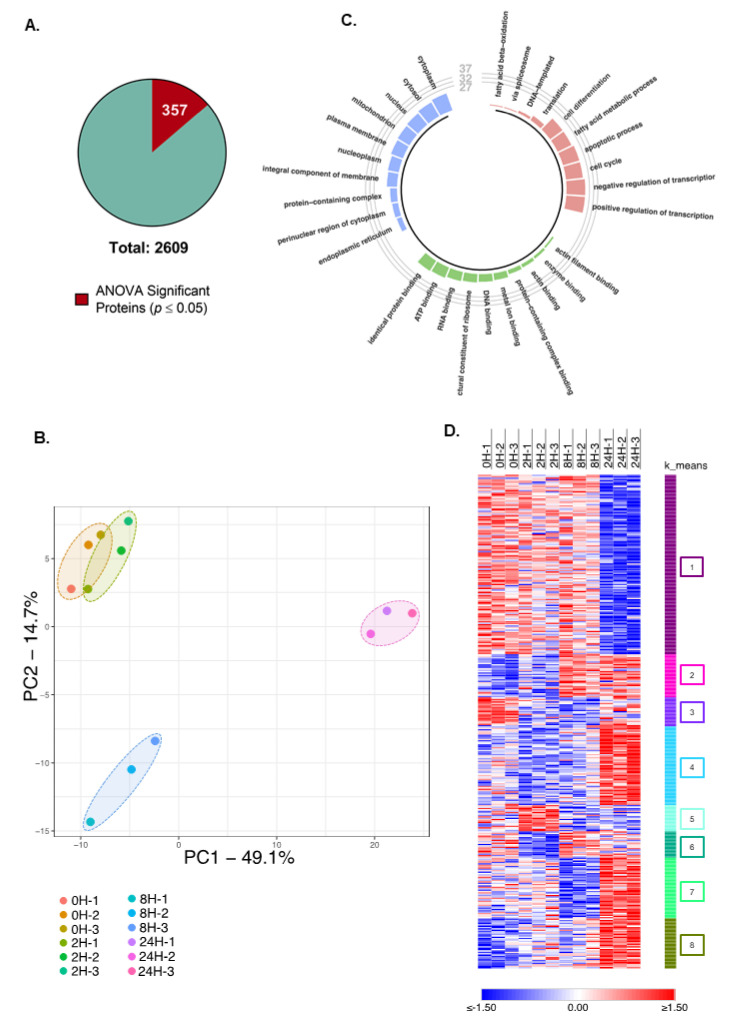
Significant proteins are distinctly regulated at the 24H time point. (**A**) Number of significant proteins compared with the total number of quantified proteins. From the total 2609 quantified proteins, 357 were significantly (*p* < 0.05) upregulated according to the ANOVA test. (**B**) PCA plot representation of each replicate used in this study based on significant proteins’ LFQ values. The PCA plot shows a clear segregation of replicates based on the treatment duration, with 8H and 24H showing the greatest distance based on PC1 and PC2, indicating distinct protein expression levels. (**C**) Circle plot representation of enriched biological processes (red), molecular function (green), and cellular component (blue) of all significantly regulated proteins. (**D**) Heat map representation of Z-scored Log2(LFQ) values of all significant proteins in each replicate. K-means clustering was used to generate clusters. Each cluster has been given a number, and they are indicated by different colors. Red hue indicates upregulated proteins, and blue hue indicates downregulated proteins.

We selected all quantified proteins that showed a significant variation in at least one-time point (*p* < 0.05) using the ANOVA test. From the 2609 quantified proteins, a total of 357 met our p-value cutoff (Figure 2A). It is important to note that these significantly regulated proteins showed a high degree of similarity in their expression levels between 0H and 2H of treatment, contrasted with 24H, which had the highest distinction among the studied time points, suggested by the relative distances in PCA plot analysis (Figure 2B). Thus, we can infer that TNFα treatment induces a gradual, yet complex regulation of the proteome of AML12 hepatocytes.

### 2.3. Exposure to TNFα Regulates Protein Synthesis and Cell Cycle Progression

TNFα is a key molecule linking obesity and insulin resistance. For the past decades, TNFα has been at the forefront of several studies investigating the patho-biochemistry of chronic, low-grade inflammation that accompanies obesity [12]. The downstream effectors and molecular consequences of the TNFα signaling pathway, and their influence in the development of insulin resistance, are well-characterized in the context of the adipose tissue [12,13,14]. Moreover, our group recently shed light on the changes in the proteome of murine adipocytes during chronic TNFα exposure, offering a glimpse into the extensive regulatory effects of TNFα at cellular and molecular levels [15]. However, the effects of TNFα on the cellular proteome of hepatocytes remain largely unexplored.

To elucidate the functional roles of the significant proteins identified in our dataset, we performed a Gene Ontology (GO) analysis [16,17] of all significant proteins simultaneously. The biological processes with highest enrichment were “positive” and “negative regulation of transcription”, “cell cycle”, “apoptotic process”, and “fatty acid metabolic process”. In accordance, most proteins were shown to be localized to the cytoplasm and nuclear cellular components, with noteworthy “ATP”, “RNA”, and “DNA binding” molecular functions (Figure 2C). These results are in line with a previous report that demonstrated TNFα induces primary murine hepatocytes to enter S-phase at 14 h of treatment [18].

To unveil the dynamics of the protein landscape of AML12 cells during TNFα exposure, we plotted a heatmap of all significant proteins across the time points studied. To characterize the expression patterns observed, the plotted proteins were clustered by k-means, in which proteins with similar regulatory profiles were clustered together. This method allows for the grouping of proteins that are likely involved in similar functional roles, thus allowing for a more thorough analysis of the underlying functional links of the identified proteins. Eight distinct clusters were observed in our dataset, numbered 1 to 8, of which cluster “1” had a considerably larger number of proteins compared with the other clusters (Figure 2D).

From the eight clusters identified in our heatmap, we decided to focus on four specific clusters that showed a clear time-dependent trend in their expression levels; namely, clusters “1”, “4”, “7”, and “8” were selected, and gene ontology was subsequently performed with the proteins from each cluster.

**Figure 3:**
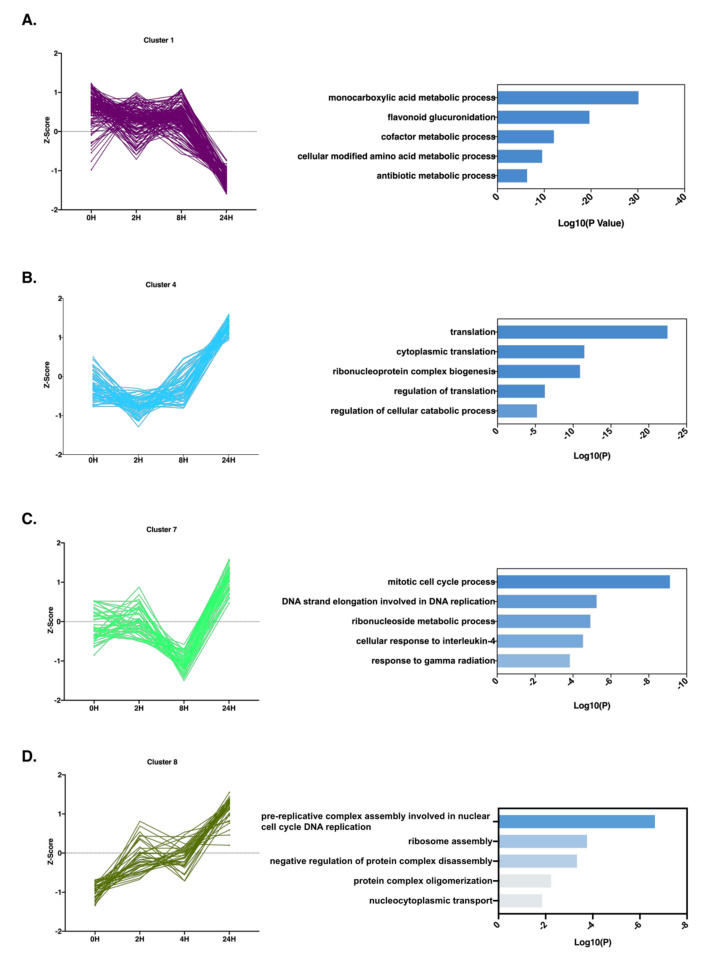
Protein clustering is correlated with protein function. (**A**–**D**) Protein dynamic regulation patterns and correspondent biological processes. Individual proteins from specific clusters were plotted based on their Z-scored Log2(LFQ) at each time point, and points were connected by a line. The top five enriched biological processes with the highest –Log(P) values are shown for the proteins present in each cluster.

Cluster “1” showed a distinct downregulation at the 24H time point, with little variation between 0H and 8H of treatment. Unsurprisingly, the proteins grouped in this cluster were primarily involved in metabolic processes, with the “monocarboxylic acid metabolic process”, “flavonoid glucuronidation”, and “cofactor metabolic process” being highly enriched (Figure 3A). Our results suggest a decrease in the activity of fatty acid catabolic pathways, which, in turn, culminate in an accumulation of FFA. Overproduction of FFA has been directly tied with deficient insulin release by pancreatic β-cells, and inhibits insulin-stimulated glucose uptake in myocytes and hepatocytes [19,20,21], leading to the development of insulin resistance and type 2 diabetes. It is worth reiterating that the downregulation of such pathways is only observed at the 24H time point, which suggests that, although FFA may enhance the insulin resistant phenotype, it is likely not the main driver for the decreased glucose sensitivity in AML12 cells, as we observe decreased uptake of glucose from as early as 2H of treatment.

The upregulation of translation is the primary pathway enriched from proteins in cluster “2” (Figure 3B). Notably, cluster “2” is mostly composed by ribosomal proteins, particularly, S and L ribosomal proteins. Our results indicate that TNFα induces protein synthesis, despite previous reports indicating an inhibitory effect of TNFα in translation initiation through the modulation of EIF-4E availability in muscle and heart cells [22]. Our data, however, show an increase in the expression levels of EIF-4G1 and EIF-4G2 at 24H compared with the 0H time point. The EIF-4G proteins associate with EIF-4E to promote ribosome recruitment and the initiation of translation [23]. Thus, TNFα in hepatocytes likely has a stimulatory, not inhibitory, effect in protein synthesis.

Although the regulatory patterns of clusters “7” and “8” are distinct, with cluster “7” showing decreased expression levels at 8H followed by overexpression at 24H, compared with the linear increase in expression levels observed in cluster “8”, both have overlapping biological processes. Clusters “7” and “8” contain proteins involved in cell cycle progression, with “mitotic cell cycle progression”, “DNA strand elongation involved in DNA replication”, and “pre-replicative complex assembly involved in nuclear cell cycle DNA replication” being the most prominent (Figure 3C,D). Among the proteins identified in cluster “7”, MCM2 together with MCM4, MCM5, and MCM6 identified in cluster “8” are of particular interest. MCM proteins (MCM2-7), which belong to the minichromosome maintenance protein family (MCM), are essential proteins in DNA replication that act as a helicase and promote replication fork progression [24]. These finding support the observation that TNFα promotes murine hepatocytes to enter S-phase and subsequently undergo mitosis.

Interestingly, clusters “2”, “3”, “5”, and “6” are also involved in DNA replication, translation, and metabolic processes (Figure S2A–D). Taken together, our data point to a proreplicative effect of TNFα, with increased nuclear protein expression involved in DNA synthesis, DNA damage repair, and cell cycle progression.

### 2.4. Time-Resolved Phosphoproteome Analysis Reveals Differential Regulation of Nuclear Proteins in Response to TNFα

Phosphorylation events are fundamental in protein signaling and key regulators in diverse cellular pathways, including glucose uptake in response to insulin [25]. TNFα disrupts the insulin signaling pathway by disrupting such phosphorylation events, specifically by inhibiting the phosphorylation of IR substrate 1, leading to a subsequent decreased glucose uptake, and eventual insulin resistance [26]. However, the activation of the TNFα signaling pathway has cascading effects that may disrupt the phosphorylation events of several downstream effectors, and may induce the phosphorylation of several target proteins. To gain a perspective on the broad effects in the phosphoproteome profile of AML12 cells in response to TNFα exposure, we conducted a time-resolved phosphoproteomic analysis of murine hepatocytes treated with TNFα subsequent to our global analysis.

**Figure 4:**
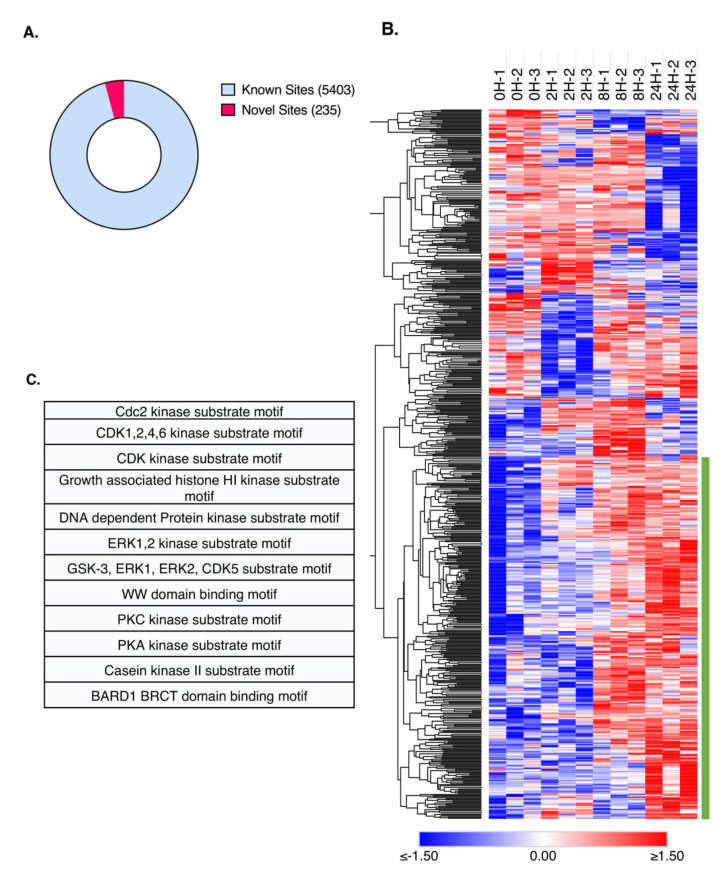
Phosphoproteomic analysis of TNFα-treated hepatocytes. (**A**) Number of novel phosphosites (red) identified in this study relative to the total number of phosphosites identified. Phosphosites were categorized as “novel sites” based on the PhosphoSitePlus database. (**B**) Heatmap representation of Z-scored Log2(LFQ) values of significantly regulated phosphosites. Significant phosphosites (*p* < 0.05 by ANOVA) were clustered based on “one minus Pearson correlation”, and the cluster of interest is highlighted by a green bar located on the left of the cluster. Red hue represents upregulated sites, while blue hue represents downregulated sites. (**C**) Substrate motif enrichment analysis of phosphosites identified in the highlighted cluster, shown in Figure 4B.

Our phosphoproteome dataset consisted of a total of 3287 proteins, mapped from 13,563 peptides, containing 12,798 phospho (STY) sites. When analyzing protein phosphorylation data, it is crucial that the modified site remains distinct, as protein regulation is site-dependent, and different phosphorylation sites often have different consequences in protein functionality and localization. Thus, for this dataset, we utilized intensity values of each phospho STY site for downstream analysis. Briefly, class I sites (sites with localization probability ≥ 0.75) were selected. We identified a total of 5638 unique class I sites, from which 235 unique phosphorylated sites are reported here for the first time (Figure 4A, Table S4). It is interesting to note that three specific consensus sequences were present in at least 500 different phosphosites across all the class I sites identified, which could suggest the preferential activation of a subset of kinases or a subset of proteins that share synergistic effects (Figure S3A). Additionally, 3825 phosphosites identified in this study were not reported in adipocytes chronically treated with TNFα in our previous study [15] (Figure S3B), suggesting an increased depth of phosphoproteome coverage in the current study (Tables S2, S4 and S5).

For downstream data processing, we conducted a statistical analysis in a similar fashion to that described in our global study. Significantly regulated class 1 phospho (STY) site intensities were considered as sites with p-values lower than 0.05 by ANOVA test. A total of 649 sites passed our criteria, and their z-score values were plotted as a heatmap (Figure 4B). A clear cluster of sites, indicated by a green stripe on the graph, showed remarkably similar phosphorylation patterns across the time points studied, in which phosphorylation events were progressively upregulated at 8H and 24H. Since this trend was the most prevalent, we hypothesize that these sites are the most likely to be regulated by TNFα treatment. We decided, therefore, to further explore these specific sites.

**Figure 5:**
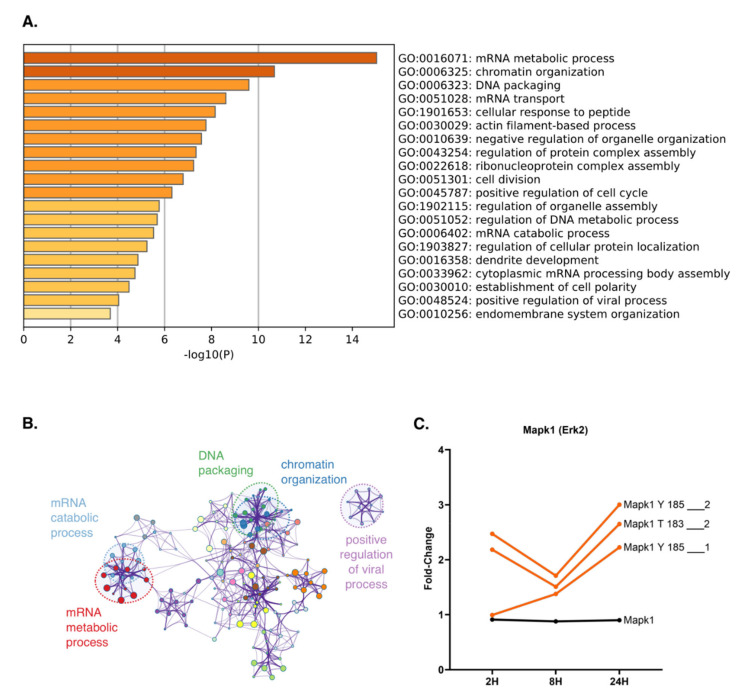
TNFα upregulates phosphorylation of proteins involved in DNA replication and cell cycle control. (**A**) Gene ontology analysis of biological processes regulated by proteins upregulated by TNFα at 24H compared with 0H. Here, analyzed proteins are represented in the cluster highlighted by the green line in Figure 4B. (**B**) String representation of functional classifications upregulated by TNFα at 24H compared with 0H. Specific clusters for relevant GO terms are observed, represented by different colors. (**C)** Fold-change analysis between site intensities at each time point relative to 0H (orange) compared with the fold-change in total protein levels at each time point relative to 0H (black). Phosphosites are labeled according to the phosphorylated residue, and its position and multiplicity.

To unveil the roles of such proteins and the cellular processes they are involved in, we performed a GO analysis, specifically focusing on biological processes. Twenty different GO terms were significantly (*p* < 0.05) enriched, the majority being related to protein transcription and translation, and cell cycle progression (Figure 5A). Among them, “mRNA metabolic processes”, “cell division”, and “positive regulation of cell cycle” are particularly noteworthy, and are in direct accordance with the biological processes enriched at the protein level. We also observed a large overlap among the enriched processes, indicated by the cluster correlation network (Figure 5B). Thus, these data further support the hypothesis that TNFα promotes cell cycle progression in hepatocytes.

Since the phosphorylation trends for phosphosites in this cluster were particularly similar, we asked if specific phosphorylation motifs were shared between such proteins. To investigate, we performed a Fisher exact test to enrich for shared motifs. We identified 12 kinase motifs significantly enriched for this cluster (Figure 4C). Of particular interest, the phosphosites in this cluster are suggested to be regulated primarily by the ERK1 and 2 kinases and their downstream effectors CDK kinases, CDK1, 2, 4, 5, and 6, which are key cell cycle regulators [27,28]. Cyclin D1 was not only significantly upregulated, but CDK4 was also phosphorylated at S300.

Although not significant, our data also revealed an increase in phosphorylation in the MAP kinases RAF1, MEK1, and ERK2. The phosphorylation of ERK2 was upregulated in two different phosphosites—specifically, Y185 and T183—while the protein levels remained unchanged throughout the course of the experiment, thus further corroborating MAP kinase activation (Figure 5C). We also mapped several phosphorylated sites in the protein Rb, which is a key regulator of cell cycle progression [29]. Taken together, our results suggest that, in hepatocytes, TNFα likely induces G1/S transition through the activation of the MAPK signaling pathway.

The promyelocytic leukemia (PML) protein was also upregulated in our data at 24H, compared with 0H of treatment. PML is a key component of subnuclear structures known as PML Nuclear Bodies (PML-NB) [30]. PML-NBs have been shown to be key regulators of several cellular processes, including responses to TNFα and IFNα [31]. Our results indicate that PML is not only upregulated at 24H of TNFα, but several phosphorylation sites were also upregulated.

### 2.5. Key Cell Cycle Regulator Proteins Are Predicted Modulators of Phosphorylation

**Figure 6:**
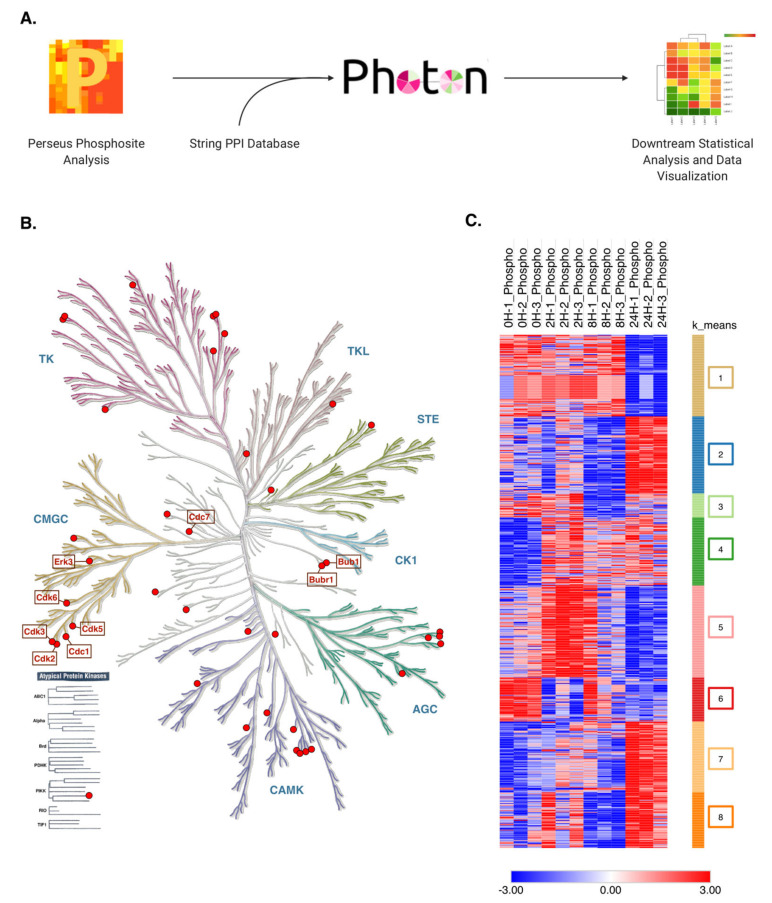
Photon software expands the analysis of proteins regulated by TNFα based on our phosphoproteomics results. (**A**) Photon analysis workflow. Processed data were used in the Photon software, using highly confident (0.9) protein–protein interactions to expand our phosphoproteomics analysis. (**B**) Photon results show that several kinases are significantly enriched based on the phosphorylated proteins identified in our results. Illustration reproduced courtesy of Cell Signaling Technology, Inc. (www.cellsignal.com, accessed on 1 September 2021). (**C**) Heatmap representation of functionality score values of significantly regulated proteins after photon analysis. Significant proteins (*p* < 0.05 by ANOVA) were clustered by k-means, and given an arbitrary color and number. Red hue represents upregulated sites, while blue hue represents downregulated sites.

Although the phosphoproteomics approach gives important insight on effector proteins and their potential functional roles, we gain little information on the secondary proteins that act as regulators and interactors, which often play key roles modulating cellular events. To expand our horizon, we utilized the PHOTON method [32] to contextualize our phosphorylation data within their signaling pathways, by utilizing STRING protein–protein interaction networks. This method allows for the identification of functional phosphosites correlated with proteins and subsequent pathway activation [32]. After filtering for class I phosphosites, our data, together with the STRING protein interaction data for *Mus musculus*, were processed with the PHOTON tool. Statistical analysis was then performed on functionality scores of the resulting output matrix. ANOVA significant (*p* < 0.05) proteins were selected for subsequent analyses (Figure 6A, Table S3).

Expectedly, several of the significant proteins enriched by the PHOTON analysis were kinases. To visualize and categorize the different kinases identified, we plotted all kinases present in our dataset in a kinome phylogenetic tree (Figure 6B). In accordance with our previous results, serine/threonine kinases from the CMGC family, which include the CDKS and ERKS, consisted of a large portion of the proteins mapped. We also observed several CAMK family proteins in our dataset. CAMK kinases, or Ca^2+^ calmodulin-dependent protein kinases, are serine/threonine kinases that have their activity modulated by intracellular levels of Ca^2+^. Upon activation, the CAM kinase-signaling cascade culminates in the phosphorylation of CaMKIV, a nuclear kinase that phosphorylates a diverse array of transcription factors. CaMKIV has been demonstrated to control a myriad of biological processes including cell cycle regulation and inflammation [33,34,35].

To shed light on the biological implications of the significant proteins enriched by PHOTON analysis, we generated a heatmap with k-means clustering to group proteins with similar functionality score patterns. We again hypothesized that proteins with similar dynamics were likely synergistic. Eight distinct clusters, numbered cluster “1” to “8”, were observed (Figure 6C). To gain a better perspective of the changes in functionality score as a function of TNFα treatment time point, all eight clusters were separately plotted and four clusters showed a clear trend in their functionality scores relative to the elapsed TNFα treatment, namely, clusters “2”, “4”, “5”, and “7”.

**Figure 7:**
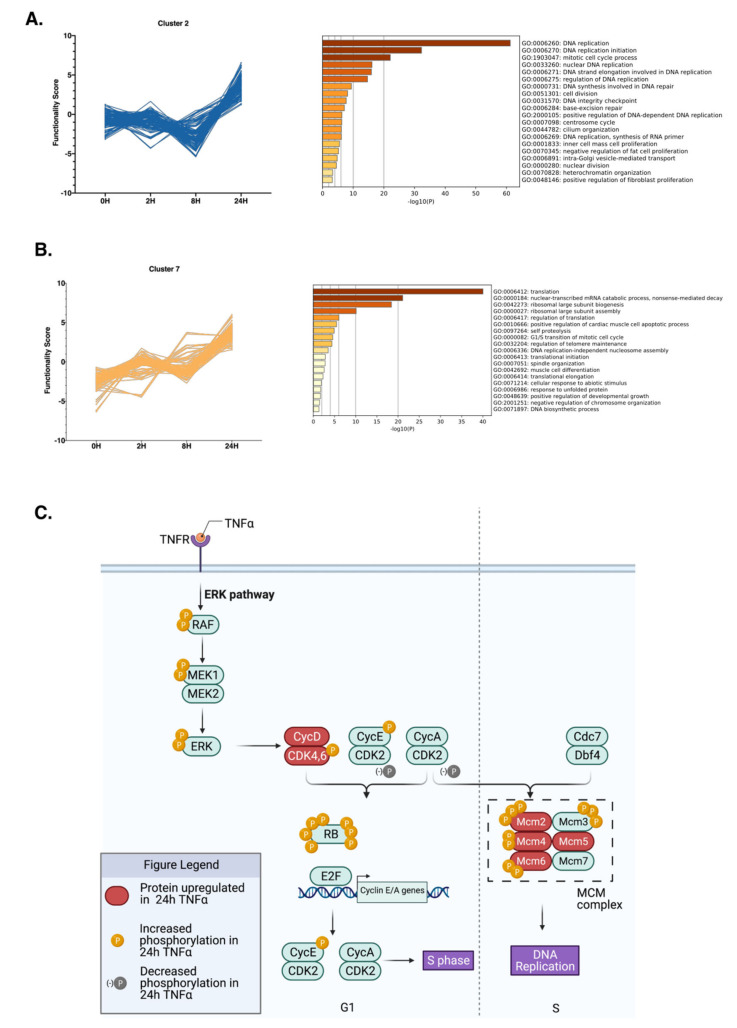
Clustered proteins show time-dependent regulation after TNFα treatment. (**A**,**B**) Proteins in clusters 2 and 7 are plotted based on their functionality score at each time point. Significantly (*p* < 0.05) regulated biological processes for these proteins are shown next to the graph. (**C**) Summary of our results. TNFα treatment induces the phosphorylation of proteins involved in the MEK/ERK kinase pathway, inducing the phosphorylation of several cell cycle regulators, including Cyclin-dependent kinases and transcription factors such as the Rb protein.

The proteins grouped in cluster “2” showed a constant functionality score between 0H and 8H of TNFα treatment, with a significant increase at 24H, indicating a late response to the TNFα exposure (Figure 7A). To unveil the cellular processes these proteins partake in, we performed a GO enrichment analysis. Most of the significantly enriched biological processes were related to cell division. Due to the comprehensive PHOTON analysis, we were able to identify a much larger number of proteins involved in each enriched pathway, and, as a consequence, the enrichment p-value was much lower, indicating a more precise result.

The biological process with the highest enrichment was “DNA replication”, which contains DNA Polymerase Alpha 1 and 2 (POLA1 and 2), Origin Recognition Complex Subunits (ORCS), MCM proteins, and several CDKs. All these proteins individually showed a significantly higher functionality score at 24H when compared with 0H of TNFα treatment (Figure S5A). “DNA synthesis involved in DNA repair” was another process that was significantly higher at 24H of TNFα exposure (Figure S5B). Furthermore, several proteins in this cluster were involved in the same pathway, and directly interacted, as evidenced by protein–protein interaction analysis (Figure S5C).

Cluster “4” was characterized by a significant increase in the functionality score of its proteins at 2H of treatment, and remain constant thereafter. This cluster had the greatest variety of enriched biological processes, ranging from “exocytosis” and “toxin transport” to “wound healing” (Figure S4A). In a similar fashion, the proteins from cluster “5” were significantly upregulated at 2H of TNFα exposure, but showed a linear decrease in their functionality scores at 8H and 24H. These proteins were primarily involved in vesicle trafficking, endocytosis, and metabolic processes (Figures S4B and S6A,B).

Proteins that constituted Cluster “7” showed a linear increase in their functionality score with respect to the duration of TNFα treatment (Figure 7B). GO analysis revealed that the most significant process enriched was “translation”, which contained mostly L ribosomal proteins, all upregulated at 24H (Figure S7A,G). Among the enriched processes, DNA replication and cell-cycle-related pathways stand out. The GO terms “G1/S transition of mitotic cell cycle”, “regulation of telomere maintenance”, “DNA replication-independent nucleosome assembly”, and “negative regulation of chromosome organization”, specifically, are very consistent with our phosphoproteome data and all proteins individually show a significant increase in their functional score at 24H compared with that at 0H of TNFα exposure (Figure S7B–F).

To verify the functional values obtained by the PHOTON method, we compared the trends of functionality scores for each of the four clusters with the Log_2_ (fold-change) of the same proteins identified in our global data. With the exception of cluster 4, most of the overlapping proteins showed a similar regulation at the protein level, as predicted by the PHOTON analysis (Figure S8A–D).

## 3. Discussion

Insulin resistance is a major health concern that is often linked to adipose tissue hypertrophy and hyperplasia, common in obese patients. In obesity, the adipose tissue often takes on dysfunctional paracrine and endocrine roles, marked by chronic secretion of pro-inflammatory adipokines, including TNFα [36]. Hepatic cells are sensitive to circulatory TNFα and can impair the canonical insulin signaling pathway, inhibiting glucose uptake and leading to the development of insulin resistance, which precedes type 2 diabetes mellitus [37,38,39]. The mechanisms underlying insulin resistance are, however, very complex, and the activation of TNFα signaling pathway has extensive ramifications on the intercellular environment that remain largely unexplored. In this study, we shed light on the dynamic changes of the hepatocyte proteome and phosphoproteome on TNFα treatment by utilizing an omics strategy.

Our results strongly indicate that cell cycle proteins are extensively upregulated by TNFα. Specifically, the expression level of these proteins increases at the 24H time point compared with the earlier time points studied, suggesting that TNFα promotes G1/S entry in hepatocytes. This finding is in line with a previous report that showed an accumulation of hepatocytes in S-phase after 14 h of TNFα treatment by flow cytometry analysis [18]. Indeed, we found many cell cycle regulators, necessary for S-phase entry, that were significantly regulated. Particularly, proteins such as proliferating cell nuclear antigen (PCNA), MCM proteins, and Cyclin-dependent kinase 4 were highly expressed at 24H. Since we used an asynchronous cell population, in which the number of cells in S-phase is roughly the same at any moment [40], the changes in the levels of these cell-cycle-dependent proteins is likely induced by TNFα treatment.

In mammalian cells, PCNA acts as a DNA polymerase processivity factor, stimulating DNA synthesis by enhancing DNA polymerase binding to DNA. PCNA expression is drastically increased at late G1 and S-phase, serving as a marker for cell cycle progression [41,42].

MCM proteins (MCM 2-7) form a protein complex that, in late G1, localize to origin recognition complex (ORC) sites on the chromatin and act as helicases, unwinding DNA and promoting the formation of replication forks, thus acting upstream of PCNA [43].

The localization and subsequent activation of MCM proteins is dictated by phosphorylation events, regulated by CDK2/Cyclin E activity [44]. Indeed, in our phosphoproteomics dataset, we were able to confirm that TNFα led to MCM family protein phosphorylation, further suggesting that TNFα acts as a driver for S-phase entry. It is important to note that while several residues of MCM proteins were significantly upregulated during the course of TNFα treatment relative to the 0H time point, they were several times greater than the changes in the global protein levels of these specific MCM proteins. Thus, the increase in the measured phosphosite is again validated (Figure S9A–D). Furthermore, consensus motifs for CDK family kinase proteins, such as CDK1, CDK2, CDK4, and CDK6 were significantly enriched in proteins phosphorylated at 24H after TNFα exposure, indicating that the phosphosites identified in our analysis are downstream effectors of the CDKs and act to promote cell cycle progression. Our results also show that ERK1 and ERK2 substrate motifs were significantly enriched, strongly suggesting that G1/S transition is driven by the MAPK signaling pathway.

Consistent with our omics results, PHOTON analysis predicted the involvement of several kinases in the activation and modulation of the identified kinases—particularly, the CMGC family kinases, of which ERK and CDK are prominent members. Similarly, the significant enrichment scores of pathways involved in DNA strand elongation and mitotic processes further support our hypothesis that TNFα not only leads to insulin resistance, but also induces cell cycle progression.

As a whole, our data suggest that TNFα promotes cell cycle progression through the activation of the MAPK/ERK pathway, which leads to the activation of cell cycle regulators. Our data reveal that TNFα exposure leads to the phosphorylation of RAF, MEK1/2, and ERK proteins. We also report overexpression and phosphorylation of cyclins and CDKs; specifically, Cyclin D and E, which stimulate the expression of Rb, hyperphosphorylated in our results, culminating in the transition from G1 into S-phase. We also identified several MCM proteins being differentially phosphorylated and upregulated in response to TNFα, reinforcing the stimulatory roles of TNFα on DNA replication and cell cycle progression (Figure 7C).

## 4. Materials and Methods

### 4.1. Cell Culture

Murine AML12 (CRL-2254) hepatocytes (ATCC, Manassas, VA, USA) were cultured in DMEM:F12 (ATCC) supplemented with FBS (ATCC), Insulin-Transferrin-Selenium and dexamethasone (Thermo-Fisher Scientific, Waltham, MA, USA). Three biological replicates (n=3) were treated with additional 10 ng/mL TNFα, for a total of 0H, 2H, 8H, or 24H [45,46,47]. At 2H before collection, medium was replaced with 10 ng/mL TNFα in serum-free medium. Cells were harvested and total cell lysate was used for proteomics analysis.

### 4.2. Metabolic Assay

Glucose uptake assay was performed using the Glucose Uptake-Glo (Promega, Madison, WI, USA). Assay was performed following manufacturer’s recommendations.

### 4.3. Protein Extraction

Cells were washed three times with 1 × PBS (4 °C), collected by scraping and subsequentially resuspended in 100 mM ammonium bicarbonate (ABC) supplemented with protease and phosphatase inhibitors. Samples were homogenized for 90 s at 6500 rpm using Precellys CK28 homogenization vials (Bertin Technologies SAS, Paris, France). Protein concentration was subsequently calculated by bicinchoninic acid (BCA) assay (Pierce Chemical Co., Rockford, IL, USA). Four volumes of cold acetone were used to precipitate 500 μg of total protein at −20 °C overnight. Samples were then centrifuged at 17,300× *g* for 15 min, supernatant was removed, and pellets were dried in a vacuum centrifuge for one min.

### 4.4. Sample Preparation for MS Analysis

Samples were fully resuspended in 50 µL of 10-mM DTT in 8-M urea and incubated in a thermomixer for 1 h at 37 °C. A total 50 µL of alkylation reagent mixture (97.5% acetonitile (ACN), 0.5% triethylphosphine, 2% iodoethanol) was added to each sample, and again incubated for 1 h in a thermomixer at 37 °C. After alkylation, samples were dried in a vacuum centrifuge and resuspended in 200 µL of 0.05 µg/uL Lys-C/Trypsin (Promega) dissolved in 25-mM ABC. Samples were transferred to a barocycler (50 °C, 60 cycles; 50 s at 20 kPSI and 10 s at 1 ATM), in which proteolysis was carried out. Peptides were desalted with the Pierce Peptide Desalting Spin Columns (Thermo Fisher Scientific, USA). A total of 20 μg of peptides from each sample were saved for global analysis. The remainder was used for phosphopeptide enrichment, performed with PolyMac spin tips (Tymora Analytical, West Lafayette, IN, USA), following manufacturer’s recommendations.

### 4.5. Mass Spectrometry Analysis

Dried samples were reconstituted in 3% ACN, 0.1% Formic Acid (FA) and separated using an Acclaim PepMap 100 C18 analytical column (75 μm ID × 50 cm) packed with 2-μm, 100-Å PepMap C18 medium (Thermo Fisher Scientific) by reverse-phase using a Dionex UltiMate 3000 RSLC coupled with the Orbitrap Fusion Lumos Tribrid Mass Spectrometer (Thermo Fisher Scientific). A 160-min gradient was used for the separation of peptides for global analysis. Sample injection was carried out using 2% mobile phase solution B (80% ACN with 0.1% FA in water). Mobile phase solution B was increased in a linear fashion until 27% B was reached at 110 min, 40% B at 125 min, and 100% B at 135 min. At this point, concentration of B was held constant for 10 min before returning back to 2% B and maintained at 2% B until the end of the run. Phosphopeptides were separated using 120-min gradient. Samples were injected at 2% B. For LC separation, solution B was increased linearly, reaching 30% B at the 80 min mark, followed by an increase of B to 45% B at 91 min and then 100% B at 93 min, at which point it was held constant for 5 min before reverting back to 2% B until the end of the run. MS analysis were performed with the orbitrap detector, with a MS1 resolution of 120,000 and MS2 resolution at 1500. Quadrupole isolation and a scan range of 375 to 1500 m/z were used. Data-dependent acquisition MS/MS was performed, with a dynamic exclusion duration of 60 s. HCD was used for fragmentation, with HCD collision energy set to 30%.

### 4.6. Protein Identification and Quantification

Raw MS/MS data were searched against the Uniprot *Mus musculus* database using the MaxQuant platform (Ver). Lys-C/Trypsin enzymes were selected for specific digestion, with 2 missed cleavage allowance. Variable modifications were set for “methionine oxidation” and for phosphoproteomics “STY phosphorylation”; “Iodoethanol” was selected as fixed modification. False discovery rate (FDR) of peptides and proteins identification was set to a standard value of 1%. Additionally, 10 ppm was selected as the main search peptide tolerance value. Peptide quantitation was performed using “unique plus razor peptides”. The proteomics results were processed and analyzed using the Perseus biostatistics platform for subsequent statistical analysis. “Contaminants”, “reverse”, and “only identified by site” proteins were filtered out, and LFQ intensity values were Log_2_ transformed. Proteins were then filtered based on 2 minimum valid values in at least one treatment group. Missing values were imputed based on the normal distribution of LFQ values. Intensity values and probability scores were used for phosphoprotein analysis. The raw phospho STY data file was filtered for proteins with a localization probability ≥ 0.75, and 2 valid intensity values in at least one time point. Missing values were again imputed with values drawn from the normal distribution. PHOTON analysis was then performed in Perseus, following the developer’s recommendations. Briefly, the STRING mouse protein–protein interaction network was imported, and only high confidence interactions (>0.9) were selected for PPI network construction. The network was then annotated with the phosphor STY site table, and the resulting signaling functionality scores were used for the downstream statistical analysis. Statistical significance was inferred based on ANOVA test. Proteins with a *p*-value ≤ 0.05 were considered significantly regulated. Gene ontology (GO) was performed using Metascape [48] online software, with only Biological Processes (GO) selected for annotation, membership, and enrichment.

## Data Availability

Raw data files can be accessed through MassIVE (https://massive.ucsd.edu/, accessed on 1 September 2021) with the ID: MSV000087248.

## Supplementary Materials

The following are available online. Figure S1: TNFα-induced regulation of glucose uptake and protein LFQ distribution. Figure S2: Protein clustering is correlated with protein function. Figure S3: Analysis of all quantified phosphosites. Figure S4: Clustered proteins show time-dependent regulation after TNFα treatment. Figure S5: Expanded view of GO biological processes highlighted in cluster 2. Figure S6: Expanded view of GO biological processes highlighted in clusters 4 and 5. Figure S7: Expanded view of GO biological processes highlighted in cluster 7. Figure S8: Protein functionality scores determined by the Photon analysis correlate with the LFQ values detected in our proteomics. Figure S9: Site-specific phosphorylation of MCM proteins is modulated by TNFα. Table S1: Global proteomics. Table S2: Phosphosites. Table S3: PHOTON analysis. Table S4: New phosphosites. Table S5: Peptide lists.

## Sample Availability

Samples of the compounds are not available from the authors.
