# Supplementary material for: Quantitative Proteomics and Phosphoproteomics Reveal TNF-α-Mediated Protein Functions in Hepatocytes
Source: Molecules. 2021 Sep 8;26(18):5472. doi: 10.3390/molecules26185472 (PMC8464716; doi:10.3390/molecules26185472)
Supplement: Supplementary file 1 [file molecules-26-05472-s001.zip › supplementary information/molecules-1314503-Supplementary.pdf]

molecules

Supplemental Data

Quantitative Proteomics and Phosphoproteomics Reveal TNF-alpha-Mediated Protein Functions in Hepatocytes

Rodrigo Mohallem [1,2] and Uma K. Aryal [1,2,*]

[1] Department of Comparative Pathobiology, Purdue University, West Lafayette, IN 47907, United States; ferreir@purdue.edu
[2] Purdue Proteomics Facility, Bindley Bioscience Center, Purdue University, West Lafayette, IN 47907, United States
* Correspondence: uaryal@purdue.edu

Supplemental Figures S1 to S9

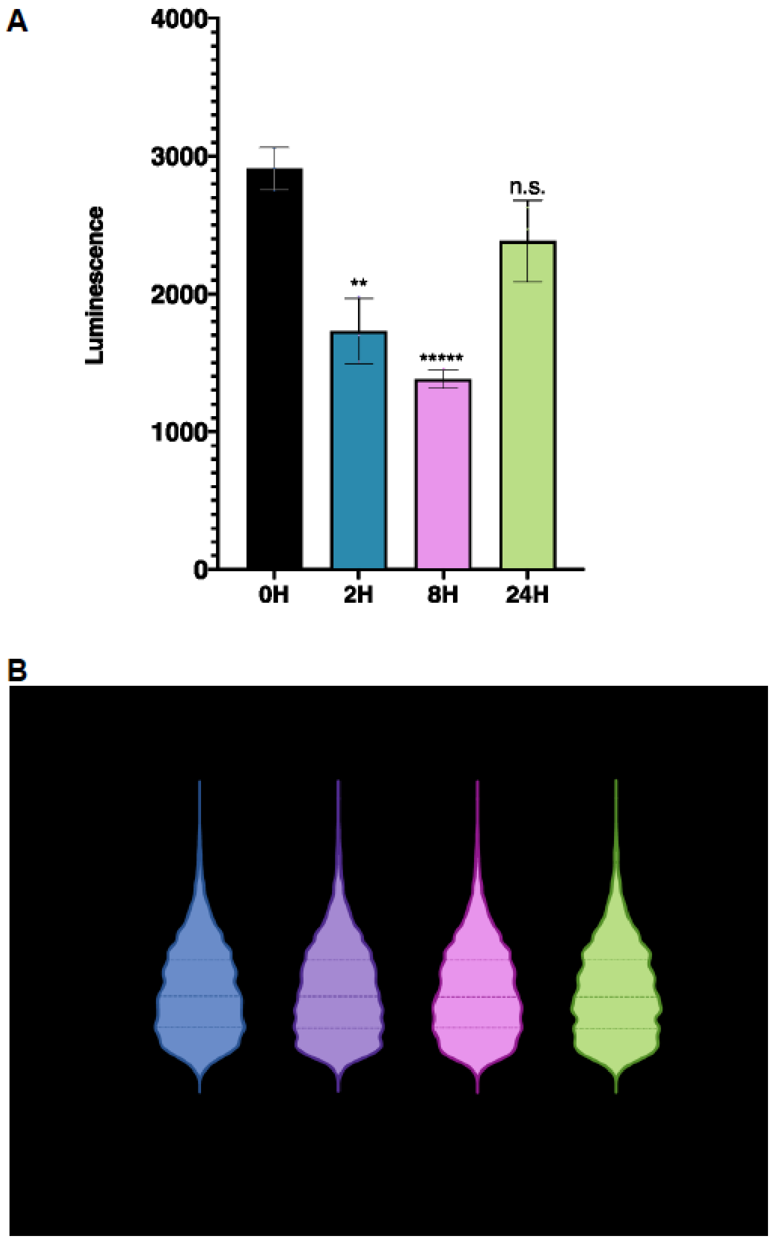

Figure S1. TNFα induced regulation of glucose uptake and protein LFQ distribution. (**A**) Glucose uptakeassay of hepatocytes treated with a single dose of TNFα for 0H, 2H, 8H and 24H. "y" axis represent raw luminescence values. (**B**) Violin plot representation of Log2 transformed LFQ values of all proteinsidentified at each timepoint analyzed.

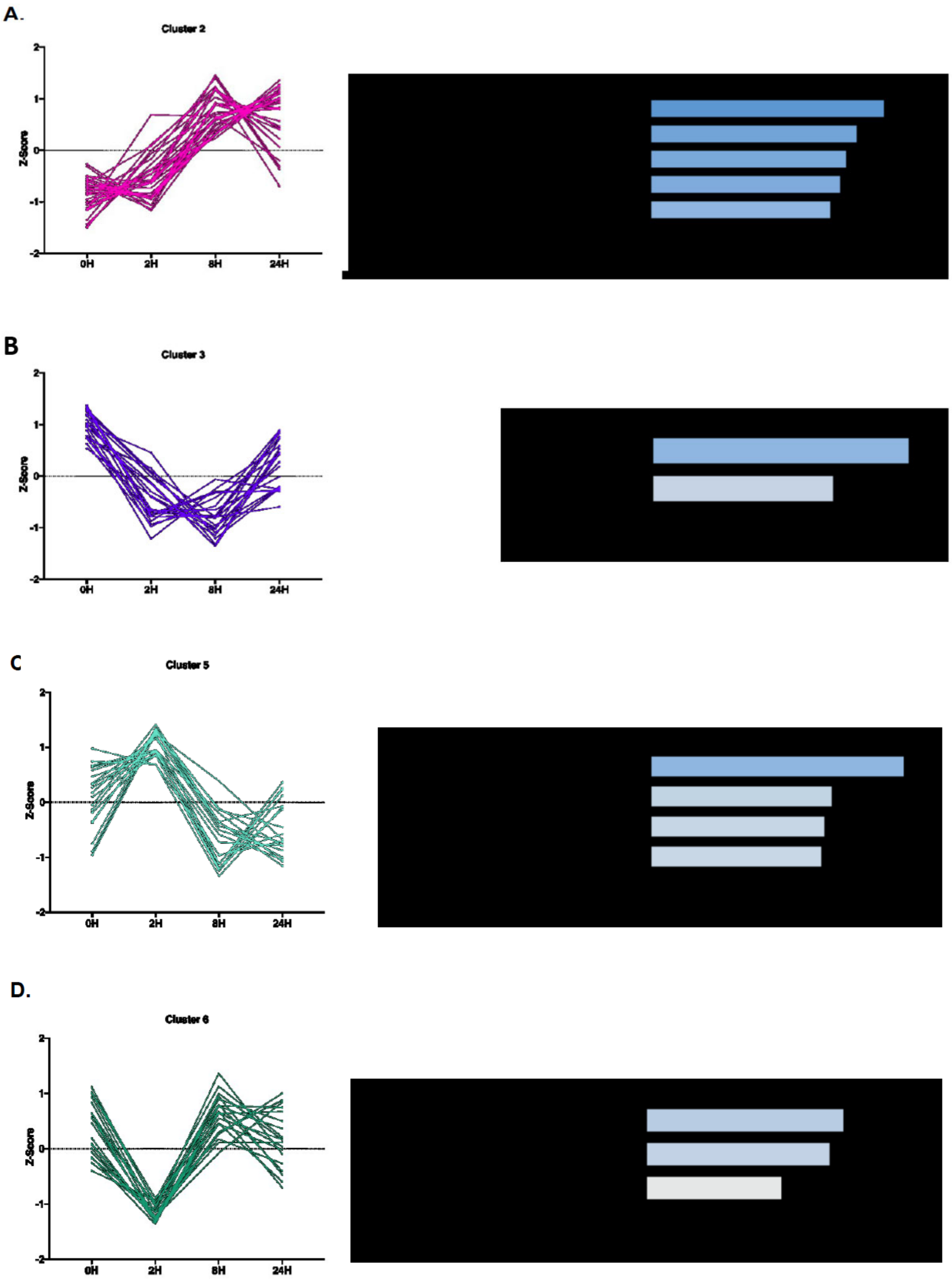

Figure S2. Protein clustering is correlated to protein function.(**A**)–(**D**). Protein dynamic regulation patterns andcorrespondent biological processes. Individual proteins from specific clusters were plotted based on their Z-scored Log2(LFQ) at each timepoint, and points were connected by a line. The top 5 enriched biologicalprocesses with highest –Log(P) values are shown for the proteins present in each cluster.

A.

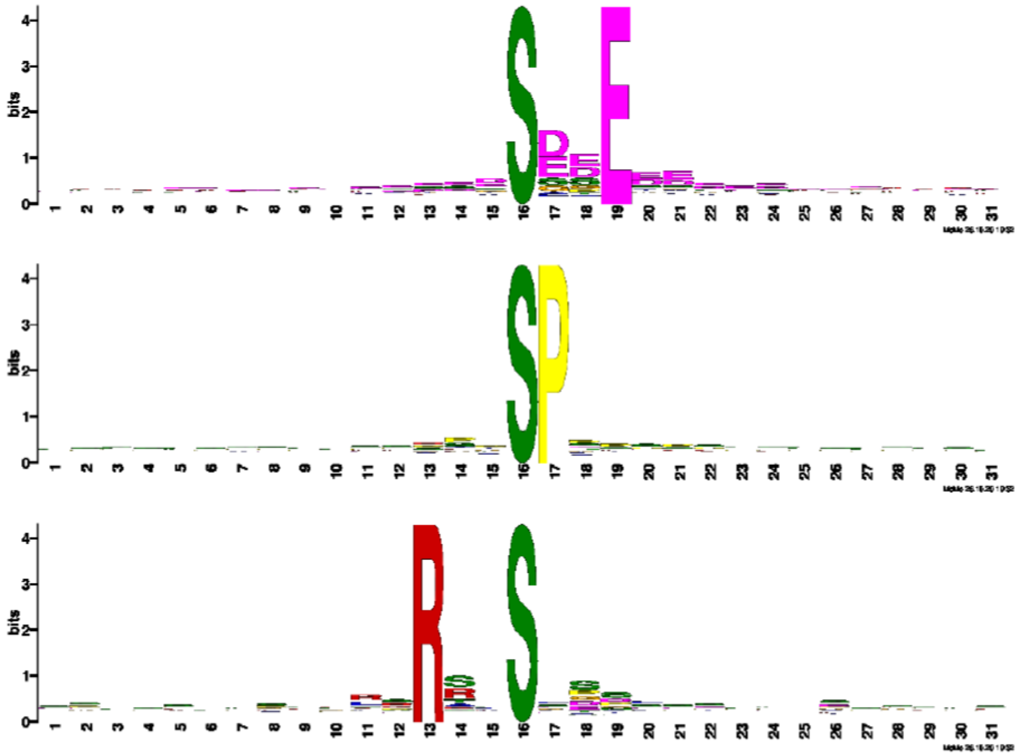

B.

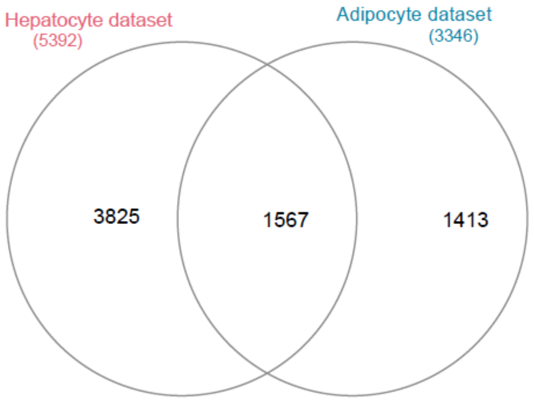

Figure S3. Analysis of all quantified phosphosites. (**A**) Sequence consensus analysis of all quantified phosphosites. Consensus sequences were determined using the sequence window for each phosphosite, andthe minimum occurrences for residue/position pair was set to 500. (**B**) Comparison of all quantified phosphosites in this study and our previous report on phosphosites identified in TNFα treated adipocytes, represented as a venn diagram. 3825 phosphosites are reported which have not been detected in our previousreport.

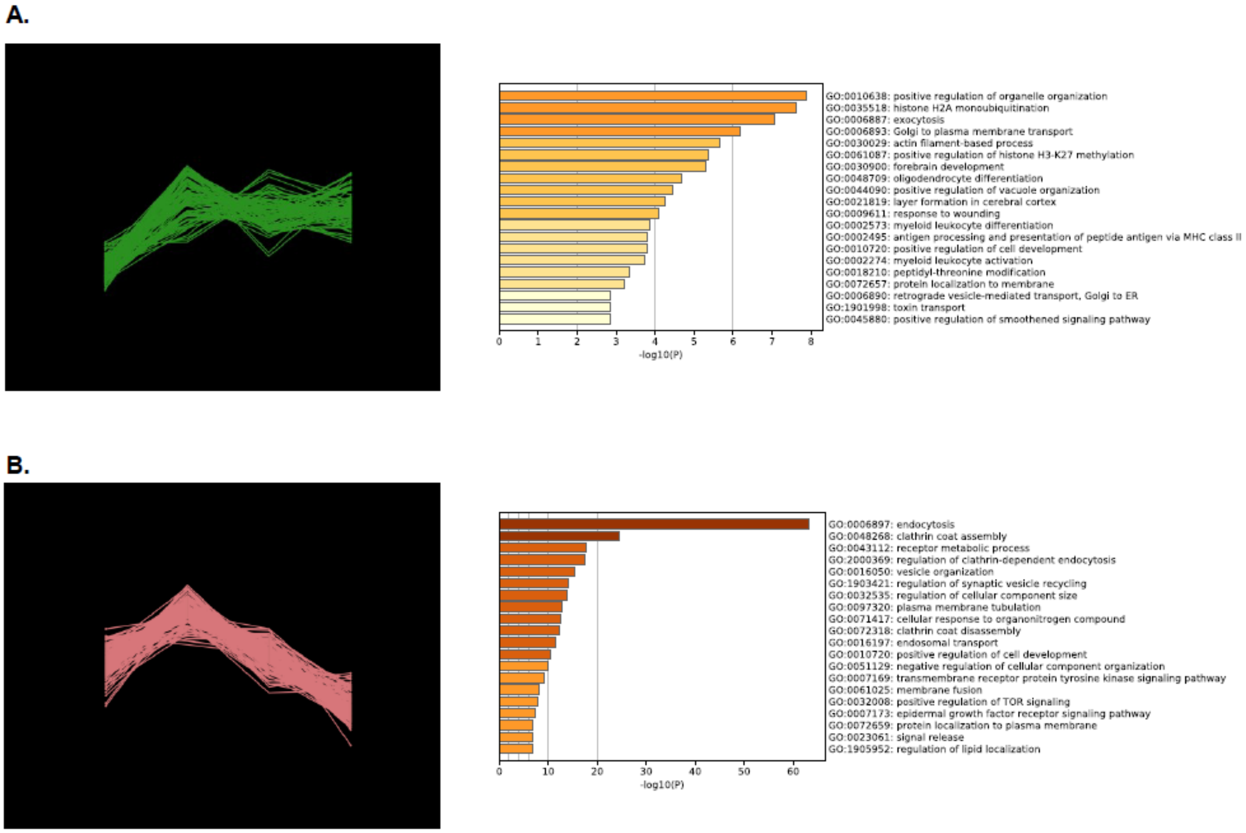

Figure S4. Clustered proteins show time dependent regulation after TNFα treatment. (**A**) and (**B**). Proteins in cluster 4 and 5 are plotted based on their functionality score at each timepoint. Significantly ($p < 0.05$) regulated biological processes for these proteins are shown next to the graph.

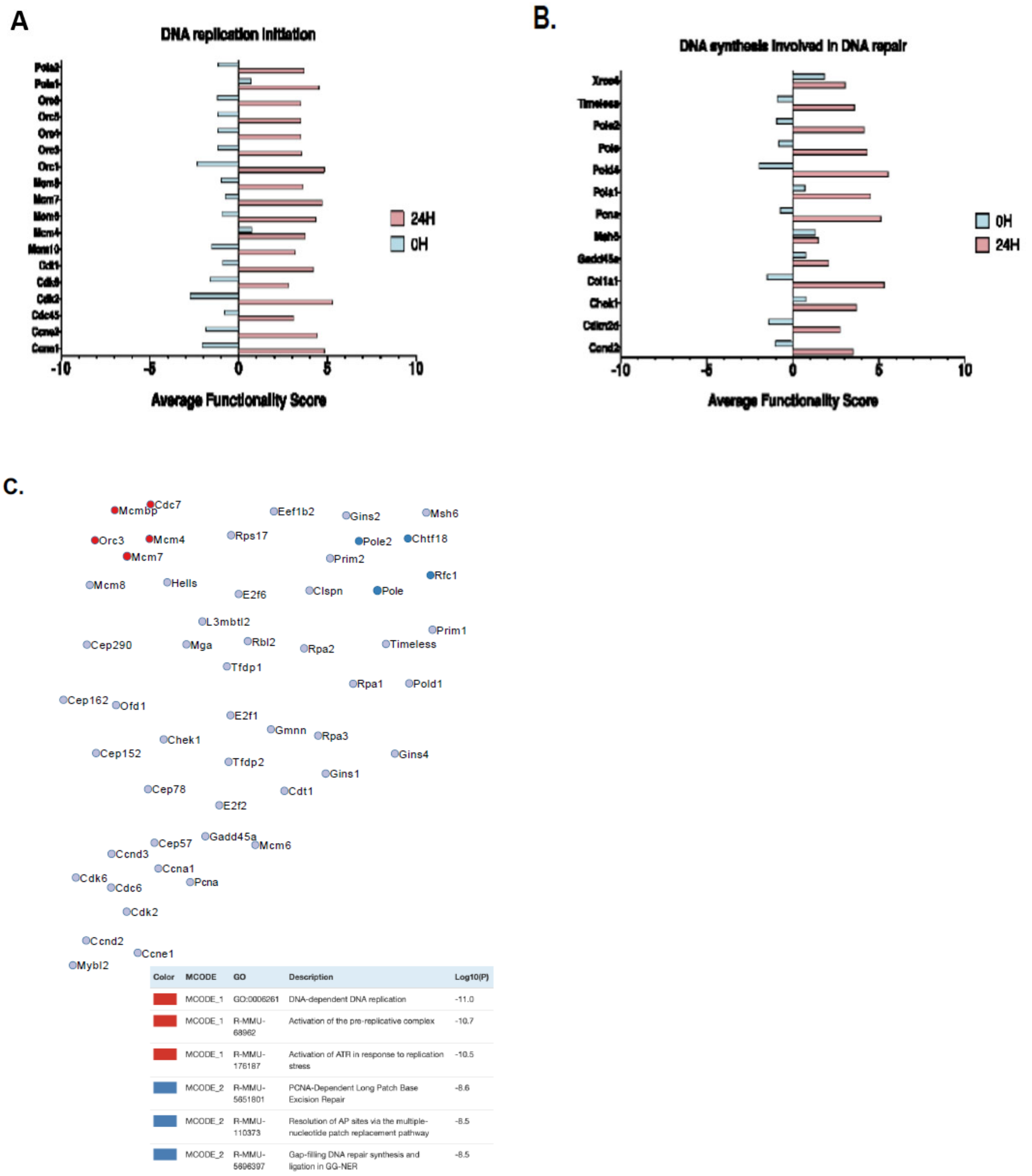

Color	MCODE	GO	Description	Log10(P)
	MCODE_1	GO:0006261	DNA-dependent DNA replication	-11.0
	MCODE_1	R-MMU-68962	Activation of the pre-replicative complex	-10.7
	MCODE_1	R-MMU-176187	Activation of ATR in response to replication stress	-10.5
	MCODE_2	R-MMU-5651801	PCNA-Dependent Long Patch Base Excision Repair	-8.6
	MCODE_2	R-MMU-110373	Resolution of AP sites via the multiple-nucleotide patch replacement pathway	-8.5
	MCODE_2	R-MMU-5696397	Gap-filling DNA repair synthesis and ligation in GG-NER	-8.5

Figure S5. Expanded view of GO biological processes highlighted in cluster 2. (**A**) Proteins involved in the biological process "DNA replication initiation". Functionality scores at 0H and 24H are shown for each individual protein. (**B**) Proteins involved in the biological process "DNA synthesis involved in DNA repair".Functionality scores at 0H and 24H are shown for each individual protein. (**C**) Protein-protein interaction network for proteins in cluster 2.

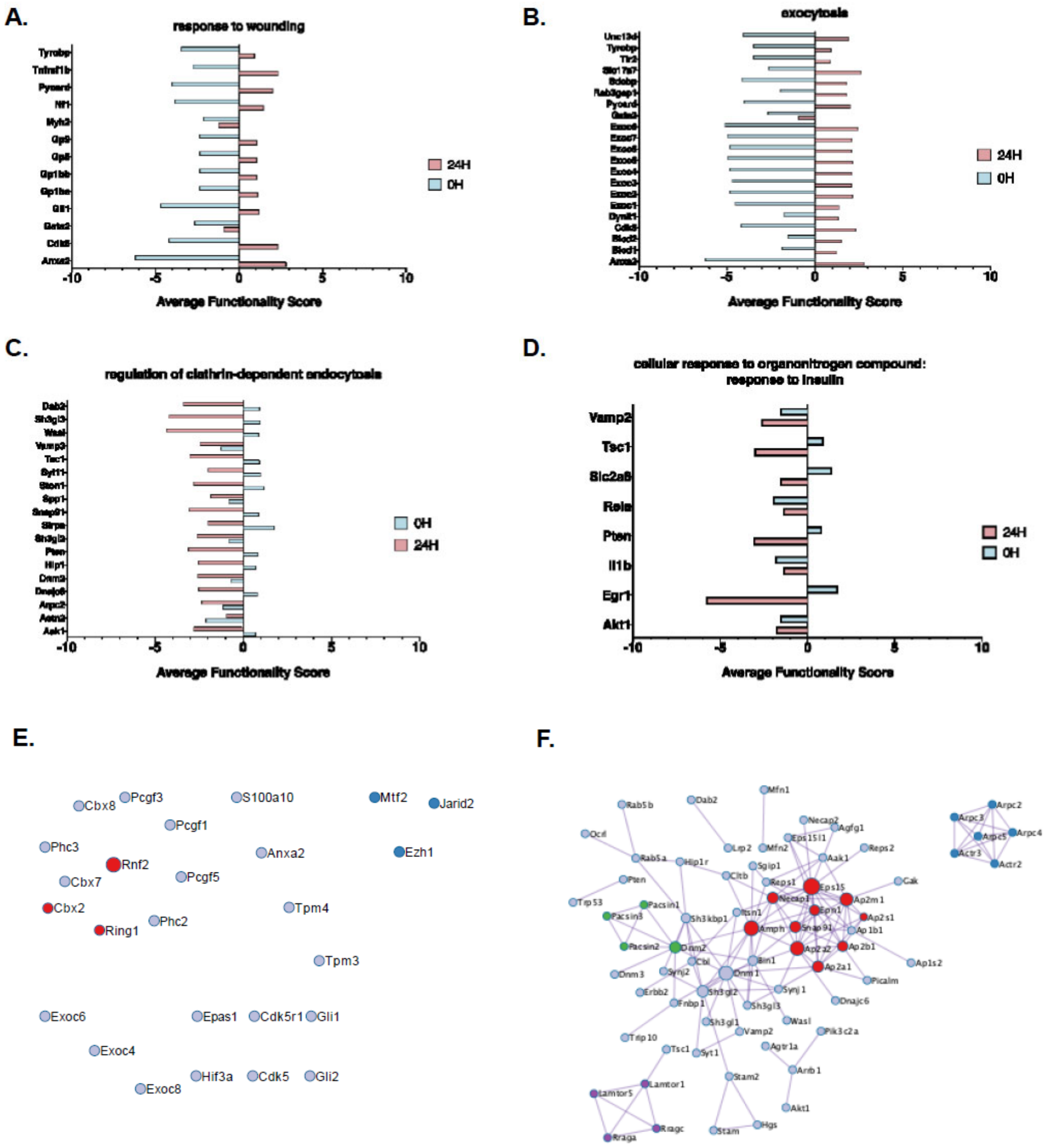

Color	MCODE	GO	Description	Log10(P)
	MCODE_1	R-MMU-8939243	RUNX1 interacts with co-factors whose precise effect on RUNX1 targets is not known	-8.4
	MCODE_1	R-MMU-3899300	SUMOylation of transcription cofactors	-8.3
	MCODE_1	R-MMU-8943724	Regulation of PTEN gene transcription	-8.1
	MCODE_2	GO:0034968	histone lysine methylation	-6.8
	MCODE_2	GO:0018022	peptidyl-lysine methylation	-6.6
	MCODE_2	GO:0016571	histone methylation	-6.5

Color	MCODE	GO	Description	Log10(P)
	MCODE_1	GO:0006897	endocytosis	-14.2
	MCODE_1	GO:0072583	clathrin-dependent endocytosis	-11.2
	MCODE_1	GO:0006898	receptor-mediated endocytosis	-9.3
	MCODE_2	GO:0034314	Arp2/3 complex-mediated actin nucleation	-16.7
	MCODE_2	GO:0045010	actin nucleation	-15.8
	MCODE_2	GO:0030838	positive regulation of actin filament polymerization	-14.1
	MCODE_3	GO:0097320	plasma membrane tubulation	-12.4
	MCODE_3	GO:0007009	plasma membrane organization	-9.0
	MCODE_3	GO:0030100	regulation of endocytosis	-7.3
	MCODE_4	GO:0032008	positive regulation of TOR signaling	-10.8
	MCODE_4	GO:0032006	regulation of TOR signaling	-9.3
	MCODE_4	GO:0031929	TOR signaling	-8.9

Figure S6. Expanded view of GO biological processes highlighted in cluster 4 and 5. (**a**) Proteins involved inthe biological process "response to wounding". Functionality scores at 0H and 24H are shown for each individual protein. (**b**) Proteins

involved in the biological process "exocytosis". Functionality scores at 0Hand 24H are shown for each individual protein. (**c)** Proteins involved in the biological process "regulation of clathrin-dependent endocytosis". Functionality scores at 0H and 24H are shown for each individual protein. (**d**) Proteins involved in the biological process "cellular response to organonitrogen compound: response to insulin". Functionality scores at 0H and 24H are shown for each individual protein. (**e**) Protein-protein interaction network for proteins in cluster 4. (**f**) Protein-protein interaction network for proteins in cluster 5.

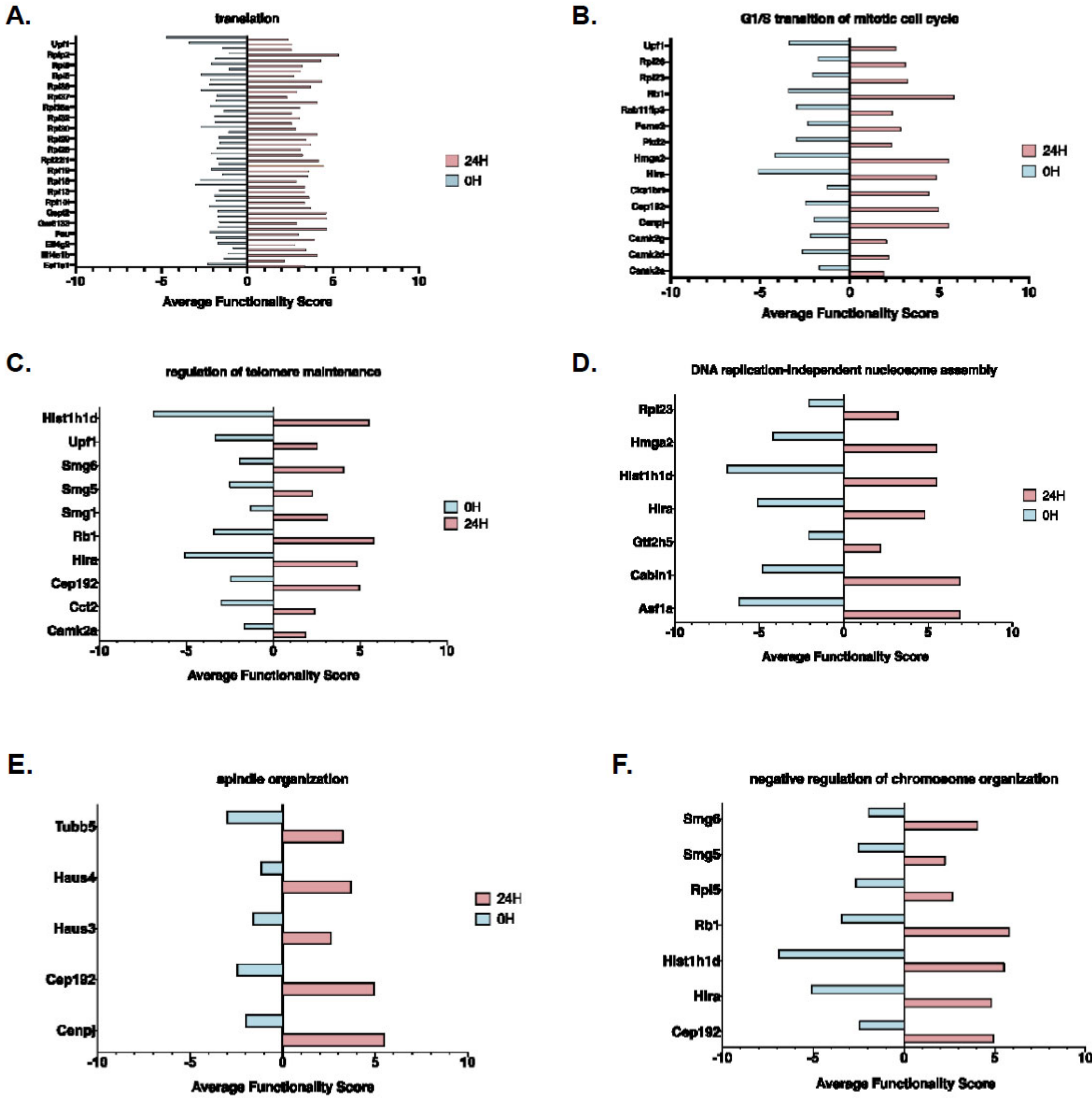
A.
translation
24H
0H
-10 -5 0 5 10
Average Functionality Score
B.
G1/S transition of mitotic cell cycle
Upf1
Rpl26
Rpl23
Rb1
Psme3
Pkd2
Hmga2
Hira
Cep192
Cenpj
Camk2g
Camk2d
Camk2a
24H
0H
-10 -5 0 5 10
Average Functionality Score
C.
regulation of telomere maintenance
Hist1h1d
Upf1
Smg6
Smg5
Smg1
Rb1
Hira
Cep192
Cct2
Camk2a
0H
24H
-10 -5 0 5 10
Average Functionality Score
D.
DNA replication-independent nucleosome assembly
Rpl23
Hmga2
Hist1h1d
Hira
Gtf2h5
Cabin1
Asf1a
24H
0H
-10 -5 0 5 10
Average Functionality Score
E.
spindle organization
Tubb5
Haus4
Haus3
Cep192
Cenpj
24H
0H
-10 -5 0 5 10
Average Functionality Score
F.
negative regulation of chromosome organization
Smg6
Smg5
Rpl5
Rb1
Hist1h1d
Hira
Cep192
24H
0H
-10 -5 0 5 10
Average Functionality Score

G.

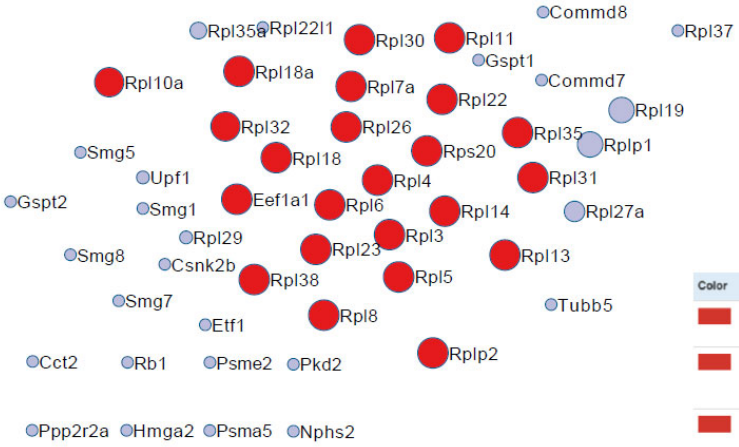

Color	MCODE	GO	Description	Log10(P)
	MCODE_1	R-MMU-1799339	SRP-dependent cotranslational protein targeting to membrane	-48.1
	MCODE_1	R-MMU-975956	Nonsense Mediated Decay (NMD) independent of the Exon Junction Complex (EJC)	-47.9
	MCODE_1	R-MMU-72689	Formation of a pool of free 40S subunits	-47.1

Figure S7. Expanded view of GO biological processes highlighted in cluster 7. (**A**) Proteins involved in the biological process "translation". Functionality scores at 0H and 24H are shown for each individual protein. (B) Proteins involved in the biological process "G1/S transition". Functionality scores at 0H and 24H are shown for each individual protein. (**C**) Proteins involved in the biological process "regulation of telomere maintenance". Functionality scores at 0H and 24H are shown for each individual protein. (**D**) Proteins involved in the biological process "DNA replication-independent nucleosome assembly". Functionality scores at 0H and 24H are shown for each individual protein. (**E**) Proteins involved in the biological process"spindle organization". Functionality scores at 0H and 24H are shown for each individual protein. (**F**) Proteins involved in the biological process "negative regulation of chromosome organization". Functionality scores at 0H and 24H are shown for each individual protein. (**G**) Protein-protein interaction network for proteins in cluster 7.

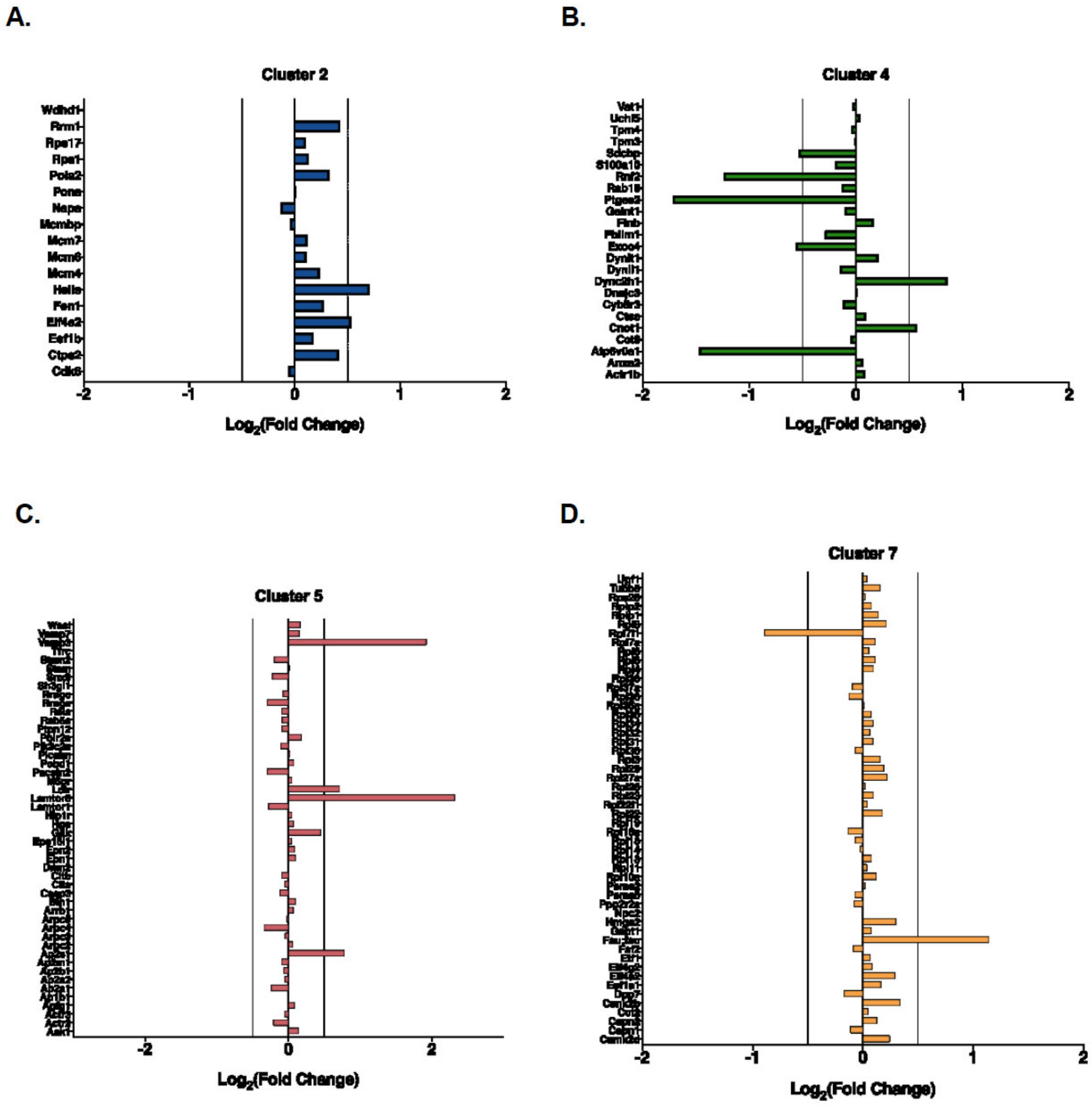

Figure S8. Protein functionality scores determined by the Photon analysis correlates to the LFQ values detected in our proteomics. (**A**) to (**D**) log2(FC) calculated in our proteomics data of proteins clustered by the photon analysis.

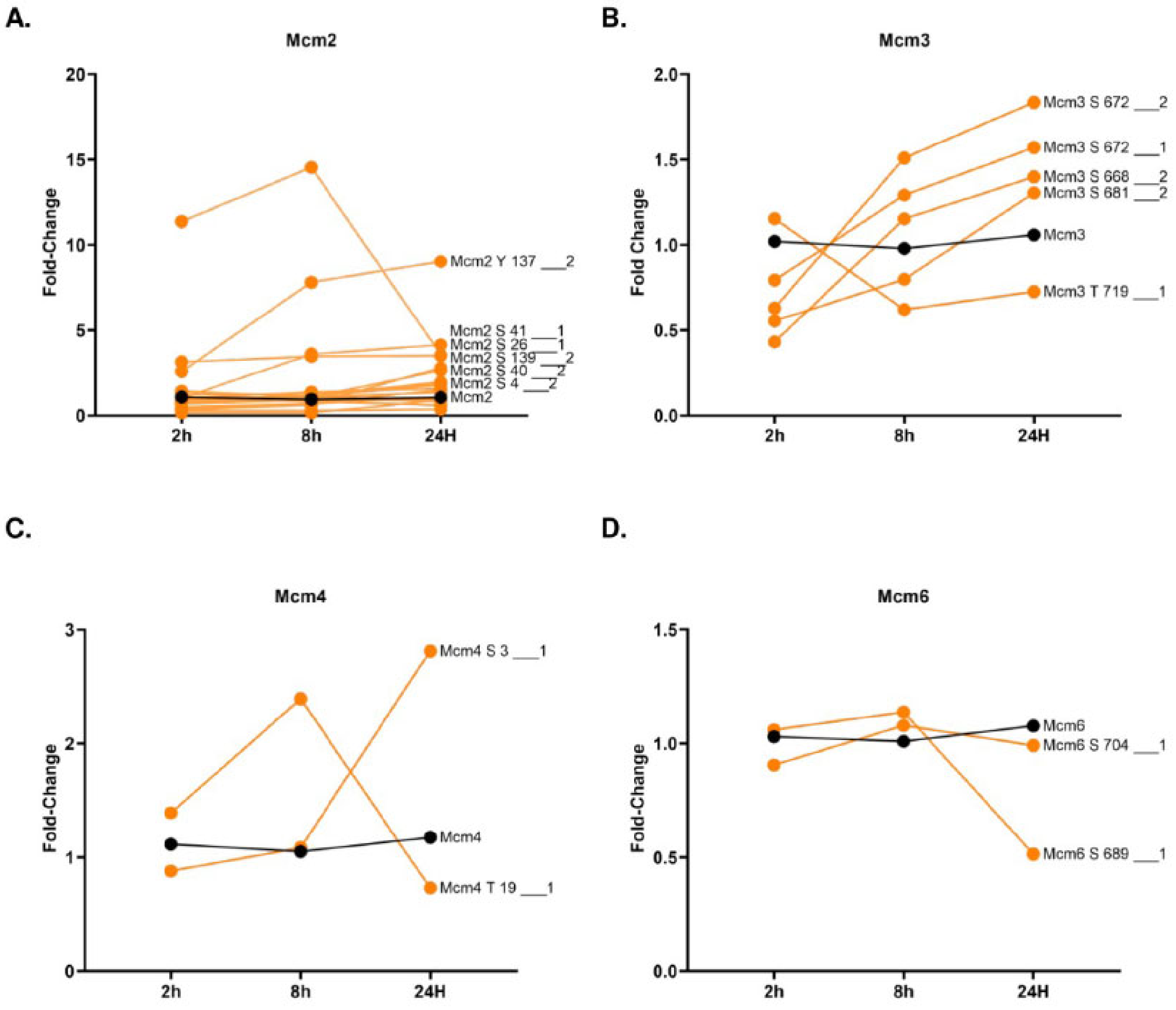

Figure S9. Site-specific phosphorylation of MCM proteins is modulated by TNFα (**A**) to (**D**) Fold-change representation of site intensities at each time point relative to 0H (orange), and fold-change representation of total protein levels at each time point relative to 0H (black) of MCM proteins identified in both phosphor and global analysis. Phosphosites are labeled according with phosphorylated residue, its position and multiplicity.
